# The Influence of Land Use Change on Landslide Susceptibility Zonation: The Briga Catchment Test Site (Messina, Italy)

**DOI:** 10.1007/s00267-014-0357-0

**Published:** 2014-08-28

**Authors:** P. Reichenbach, C. Busca, A. C. Mondini, M. Rossi

**Affiliations:** 1 Consiglio Nazionale delle Ricerche, Istituto di Ricerca per la Protezione Idrogeologica, Perugia, Italy; 2 Dipartimento di Scienze della Vita e dell’Ambiente, Università Politecnica delle Marche, Ancona, Italy

**Keywords:** Shallow landslide, Land use change, Susceptibility models and zonation, Satellite images

## Abstract

The spatial distribution of landslides is influenced by different climatic conditions and environmental settings including topography, morphology, hydrology, lithology, and land use. In this work, we have attempted to evaluate the influence of land use change on landslide susceptibility (LS) for a small study area located in the southern part of the Briga catchment, along the Ionian coast of Sicily (Italy). On October 1, 2009, the area was hit by an intense rainfall event that triggered abundant slope failures and resulted in widespread erosion. After the storm, an inventory map showing the distribution of pre-event and event landslides was prepared for the area. Moreover, two different land use maps were developed: the first was obtained through a semi-automatic classification of digitized aerial photographs acquired in 1954, the second through the combination of supervised classifications of two recent QuickBird images. Exploiting the two land use maps and different land use scenarios, LS zonations were prepared through multivariate statistical analyses. Differences in the susceptibility models were analyzed and quantified to evaluate the effects of land use change on the susceptibility zonation. Susceptibility maps show an increase in the areal percentage and number of slope units classified as unstable related to the increase in bare soils to the detriment of forested areas.

## Introduction

The spatial distribution of landslides is the consequence of different climatic situations and environmental settings, including topography, morphology, hydrology, lithology, and land use conditions. In slope stability analysis, lithology and geological structure can be considered constant over long periods whereas morphology, climate, and land use can be affected by major modifications seasonally or over a period of decades. Changes in land use distribution and type can be natural or induced and controlled by human actions. Recent studies focusing on the effect of human-induced land use changes on slope stability have shown that in populated regions, the impact of humans on the environment contributes significantly to the initiation and reactivation of landslides (e.g., Vanacker et al. 2003; Meusburger and Alewell 2008; Van Den Eeckhaut et al. 2009; Bruschi et al. 2013). It well known that different land use types may control the stability of slopes, and in particular, slope stability is enhanced by vegetation in terms of mechanical and hydrological characteristics (Greenway 1987).

For single slopes, many studies have evaluated in detail how the architecture and the distribution of the plant root system can strongly influence the stability (Stokes et al. 2008; Mao et al. 2014). At small scale, the influence of the spatial distribution of different land use types on slope stability has been evaluated using different techniques: Yi et al. (2010) have presented a case study in Enshi (China) where human action and the cultivated areas (mainly dry land, rice field, and terrace) play an important role in accelerating slope weathering and instability processes. Glade (2003) described examples from different parts of New Zealand that indicate changes in sediment-generating processes following land use modifications. After deforestation, landslides contributed significantly to sedimentation sequences in depositional basins such as lakes, swamps, estuaries, coastal wetlands, and the near shore and offshore zones of continental platforms. Van Beek and Van Asch (2004) applied a physically based model to a 1.5 km^2^ catchment in the Alcoy region (SE Spain) to evaluate the effects of land use change on the spatial and temporal activity of slope instability. These authors observed that the abandonment of cultivated fields induces a significant decrease in land slipping frequency, and in sediment delivery. Karsli et al. (2009) examined the relationship between the number of tea gardens in Turkey and landslide density. The land cover change caused an increase in the landslide occurrence, causing more severe property damages and casualties. Vanacker et al. (2003) proposed a methodology to investigate the effect of land use/land-cover change on slope movement susceptibility by incorporating specific hydrologic parameter estimates, in a simple process-based slope stability model. The analyses confirmed the hypothesis that the overall susceptibility to slope movement is highly dependent on recent land use change. In particular, the conversion from secondary forest to grassland and/or cropland increased the occurrence of shallow slope movements.

In this work, we evaluated the influence of land use change on the spatial distribution of landslide occurrence (susceptibility) at the basin scale for a study area located in the Briga catchment.

Landslide susceptibility (LS) was described by Brabb (1984) as the likelihood of a landslide occurring in an area on the basis of local terrain conditions, and can be defined as the degree to which an area can be affected by future slope movements (Guzzetti et al. 1999, 2005, 2006a, b). Several LS zonations have been proposed in the literature using different techniques and models, numerous combinations of thematic variables, and various methods to evaluate the model fitting performance and the prediction skills. To evaluate the effect of land use distribution on LS, we have exploited two different maps portraying the 1954 and the 2009 land use distribution and some scenarios obtained by changing the pattern and the distribution of the land use classes of the 2009 map. Using the 2009 land use distribution and a set of morphological information, we have prepared LS zonation exploiting different multivariate statistical classification techniques. To analyze the effect of land use change, we have applied the derived models in the same area considering the land use distribution obtained from one aerial photograph taken in 1954.

## Study Area

**Fig. 1:**
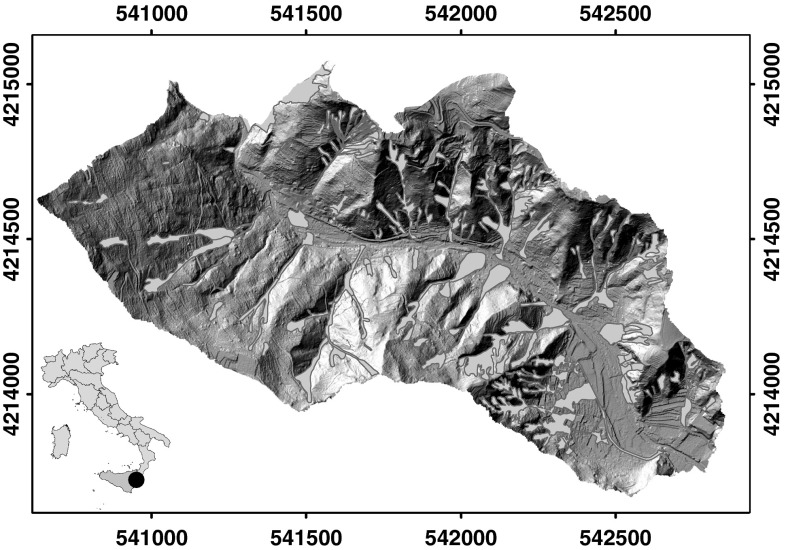
Shaded relief of the study area located in the Briga catchment, along the Ionian coast of Sicily (Italy). *Red polygons* show landslides triggered by the October 1, 2009 rainfall event (Color figure online)

The study area where we analyzed the influence of land use change on LS (1.7 km^2^) is the eastern part of the Briga catchment (Fig. 1) located in the Messina province, along the Ionian coast of Sicily (Italy). The Briga catchment is situated along the eastern-facing slope of the Peloritani Mountains, where nappes of the upper internal complex (Kabilo-Calabride Units), consisting of metamorphic rocks, crop out (Carbone et al. 2008). In the study area, elevation ranges from sea level to about 500 m, and terrain gradient is in the range of 0°–81°. The catchment exhibits an ephemeral hydrological regime. Climate is Mediterranean with hot and dry summers, and precipitation falling mostly in the period from October to January. Landslides, including shallow soil slides and debris flows, deep-seated rotational and translational slides, and complex and compound failures, are abundant, and caused primarily by rainfall (Goswami et al. 2011).

On October 1, 2009, the Briga catchment and the surrounding area for a total extent of about 60 km^2^ were hit by an intense storm with more than 220 mm of rain in 7 h with peak of 10.6 mm in 5 min measured at the St. Stefano di Briga rain gauge (Maugeri and Motta 2011). The rainfall event triggered more than 1,000 shallow landslides, mainly shallow soil slides and debris flows. Landslides and inundation caused 37 fatalities, numerous injured people and severe damages in the affected villages and along the transportation network.

## Available Data

### Landslide Inventory Map

After the event, a detailed landslide inventory map at 1:10,000 scale was prepared for the entire Briga catchment (Ardizzone et al. 2012). The inventory was obtained through a combination of: (i) field surveys carried out in the period from October to November 2009, and (ii) visual interpretation of pre-event and post-event stereoscopic and pseudo-stereoscopic aerial photographs. The inventory map shows: (i) the distribution and types of the event landslides triggered by the October 1, 2009 rainfall event (Fig. 1), and (ii) the distribution and types of the pre-existing landslides. Landslides were classified based on the prevalent type of movement (Cruden and Varnes 1996), the estimated depth, and their relative age.

Landslides triggered by the October 1, 2009 rainfall event were mapped through the visual interpretation of pseudo-stereoscopic color photographs taken shortly after the event at 1:3,500 scale, and digital stereoscopic photographs taken in November 2009 at approximately 1:4,500 scale. The event triggered mostly shallow, composite soil slide–debris flows, and shallow slides. Soil slide–debris flows occurred isolated or clustered in groups of several failures, and affected open slopes and low-order drainage channels. Most of the soil slides occurred on steep slopes where the material was completely mobilized, leaving empty scars. In the study area, we mapped more than 147 shallow soil slides for a total extent of about 0.14 km^2^. Landslide source areas occurred mostly in “no vegetation” zones (66.6 %), in pasture (15.8 %), or in forest (14.7 %), whereas only a very small portion affected urban (1.0 %) and cultivated areas (1.9 %).

The pre-event landslides were mapped through the visual interpretation of 1:33,000 scale stereoscopic black and white aerial photographs flown in 1954 by the Istituto Geografico Militare. Pre-existing landslides are represented by rock falls, topples, debris flows, slides, and complex landslides. In the study area, pre-event landslides are mainly slides. Small slides are mostly shallow translational movements located inside other landslides and along undisturbed slopes. Large slides are deep-seated, rotational, and translational slides with a well-defined depletion zone characterized by a concave profile with multiple vertical escarpments and trenches, and a distinct bulging deposit characterized by an irregular or convex profile (Ardizzone et al. 2012).

### Land Use Maps

**Fig. 2:**
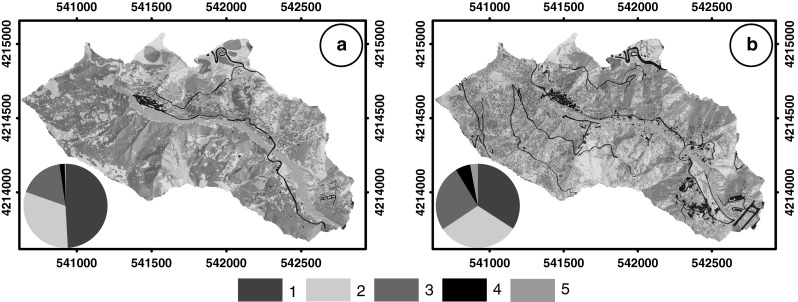
Land use maps obtained by **a** aerial photographs acquired in 1954 and **b** recent QuickBird images. *1* forest, *2* pasture, *3* no vegetation, *4* urban area, *5* cultivated area. *Pie charts* show the distribution of classes in the two maps

For the study area, two maps reporting the land use in different periods were prepared exploiting available aerial photographs and very high resolution (VHR) satellite imagery. The first map (Fig. 2a) was derived from the analysis of the same black and white aerial photograph taken in 1954 that we used to map pre-event landslides. The second map (Fig. 2b) was obtained from a QuickBird satellite imagery bundle (one 0.6-m ground sample distance panchromatic band and four 2.4-m ground sample distance multispectral bands) taken on September 2, 2006 and further verified through a Quickbird bundle taken on October 8, 2009, a few days after the event. Both satellite images were pan sharpened in a multispectral bundle at 0.6-m ground resolution and orthorectified (Mondini et al. 2011).

To prepare the 1954 land use map (Fig. 2a), we digitized the aerial photograph with 16 bits relative radiometric resolution and 1200 dot/in. scan resolution. We orthorectified the digitized image through the *i.ortho.photo* GRASS module (release 6.4.1.), specific for aerial photographs, using a high-resolution DEM, and 8 Ground Control Points (GCP) chosen from the satellite image (Rocchini et al. 2012) and a bilinear interpolation. The high-resolution DEM (1 m × 1 m) was obtained a few days after the rainfall event (October 5–7, 2009), by the Italian national Department for Civil Protection that flew in the study area with an airborne Lidar sensor. The co-registration error (RMSE) with the satellite image is about 4 m in both *x* (east) and *y* (north) directions. We classified the orthorectified image following three main steps: (i) growth of seeds (see below) representing selected land cover classes, (ii) manually interpreting and contouring of areas not selected in the first step and (iii) post-processing. The first step requested a preliminary visual interpretation of the scene to identify the main land cover types in the study area. Due to the poor spectral content of the image, we recognized only four main land use classes: forest, pasture, bare soil, and urban area. For each class, we selected seeds or clusters of representative points and then we grew up the seeds on the basis of specified spectral distance thresholds (ENVI 4.8 user’s guide, 301–302). We selected the thresholds depending on the homogeneity of the areas around the seeds. We chose conservative thresholds (standard deviation ranging from 0.5 to 2) and we classified the remaining areas through a manual contouring. To remove small clusters of unclassified points, or singular points (“salt & pepper” effect), we sieved and clumped (ENVI 4.8 user’s guide, 655–657) the classification through a 3 × 3 kernel. We checked the accuracy of the classification through the direct comparison between homologous points in the classified image and in the original aerial photograph. The accuracy of the classification in terms of correct percentage of class membership attribution is listed in Table 1.

**Table 1:** Areal extent (in %) of the land use classes (see pie charts in Fig. 2) and accuracy of the classification for each class for the two land use maps

Land use classes	Extent (%)			Accuracy (%)	
	1954 map	2009 map	Difference	1954 map	2009 map
Forest	49.41	34.14	−15.27	90	94.1
Pasture	31.25	31.49	0.24	86.4	80
Bare soil	17.32	25.70	8.38	88.6	69
Urban area	2.02	5.73	3.71	90.9	100
Cultivated area		2.94	2.65	NA	77.7

To prepare the 2009 land use map (Fig. 2b), we exploited two satellite images. We first classified the image acquired in 2006 by using the maximum likelihood classifier (Richards and Jia 1999) and five training area sets corresponding to: forest, pasture, bare soil, urban area, and cultivated land cover classes. Cultivated areas were not recognized in the 1954 scene. We post-processed the obtained classification through sieving and clumping operations. We qualitatively verified that no relevant land cover changes occurred between the classification obtained by the image acquired in 2006 and the land cover shown in the 2009 image. For this reason, we assumed that this map is representative of the area land cover of the 2009 rainfall event. The choice of classifying the image acquired in 2006 rather than the 2009 image is due to the widespread presence of the landslides triggered by the event. The land use classification was validated through the direct comparison between homologous points in the classified and in the original images. The accuracy of the classification is reported in Table 1.

### Slope Units

To prepare the LS zonation, we have partitioned the study area into slope-units (SU), hydrological terrain subdivision bounded by drainage and divide lines (Carrara et al. 1991). The SU were outlined exploiting a 5-m resolution DEM obtained resampling the VH resolution DEM provided by the Italian national Department for Civil Protection and using standard WPS (Web Processing Service) tools based on GRASS GIS 7 (grass.osgeo.org/grass70/), R (www.r-project.org/), and Python Web Processing Service (PyWPS) (Marchesini et al. 2012). The size and the geometrical characteristics of the SU are controlled by modeling parameters defined by the user including the minimum half-basin area (http://grass.osgeo.org/grass64/manuals/r.watershed.html) and the slope aspect variability described as the standard deviation of the aspect sine and cosine. In the study area, the procedure identified 238 slope units which represent the mapping units of reference for the determination of LS. The dimension of the SU ranges from 1,348 to 28,341 m^2^ with a mean value of 7,194 m^2^. For each slope-unit, we calculated descriptive statistics of elevation and slope that were used as variables to explain the spatial distribution of landslides (Carrara et al. 1991, 1995).

## Landslide Susceptibility Zonation

### Single and Combined Models

To evaluate the influence of land use change on LS, we have prepared several zonations through different multivariate statistical analyses of morphologic and land use information. The percentage of event landslides in the slope units was used as the dependent variable (grouping variable) for the terrain classifications. Following previous experiences in modeling LS (Carrara et al. 1991, 1995, 1999; Guzzetti et al. 1999), slope units having less than 2 % of the area covered by slope failures were considered free of landslides, and slope units having more than 2 % of their area covered by slope failures were considered as containing landslides. Adopting this approach, 114 slope units were identified as having landslides, and 124 were considered free of landslides.

Exploiting the presence or absence of event landslides and the thematic information, we have prepared three single and one combined LS zonation following the approach described by Rossi et al. (2010). Single susceptibility zonations were obtained with different multivariate classification techniques (Michie et al. 1994), including: (i) linear discriminant analysis (LDA) (Fisher 1936; Brown 1998; Venables and Ripley 2002), (ii) quadratic discriminant analysis (QDA) (Venables and Ripley 2002), and (iii) logistic regression (LR) (Cox 1958; Brown 1998; Venables and Ripley 2002). For the combined approach (CM), we adopted a regression schema where the presence or absence of event landslides in each slope unit was taken as the dependent variable and the results of the single models were the independent, explanatory variables (Rossi et al. 2010; Clemen 1989).

For the multivariate terrain classifications, we exploited the percentage of the 2009 event landslides above a selected threshold as grouping variable and six morphological, five land use classes obtained from the 2009 land use map and the presence of pre-event landslides as explanatory variables. The categorical explanatory variables (land use classes and pre-event landslides) for the multivariate terrain classification were computed in a GIS as the percentage of each class in each slope unit. Morphological variables were obtained from the same DTM used to perform the subdivision of the study area into slope units and included statistics of the elevation and of the slope.

**Fig. 3:**
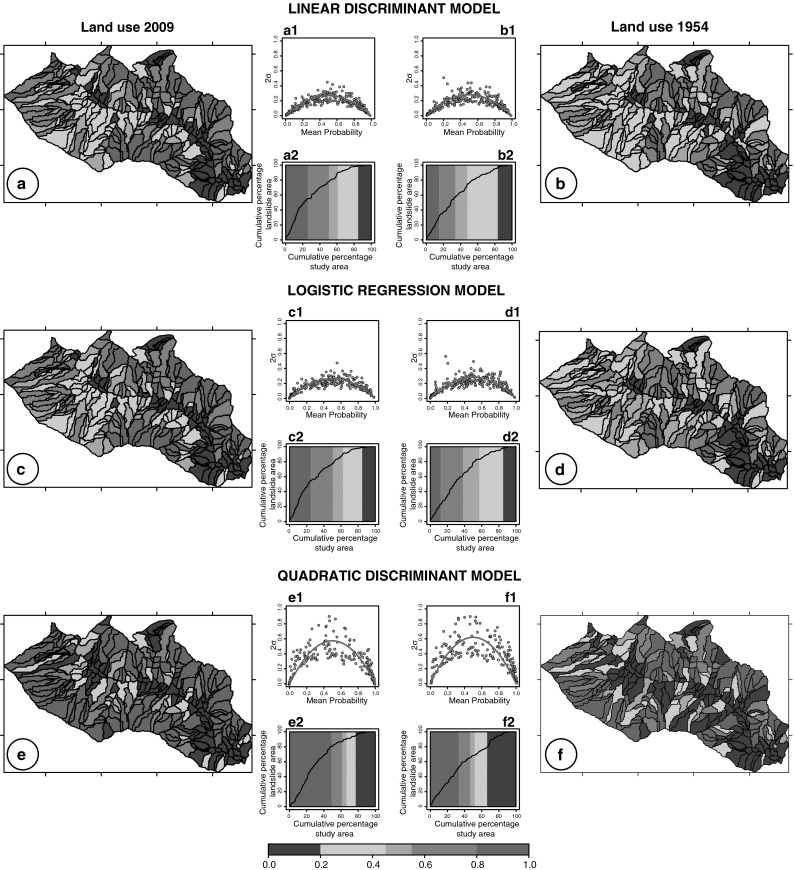
Results of single landslide susceptibility models (2 % threshold). On the right models prepared using the 2009 land use (**a**, **c**, **e**) and on the left the 1954 land use map (**b**, **d**, **f**) with the predicted LS in five unequally spaced classes; (*a1*, *b1*, *c1*, *d1*, *e1*, *f1*) plot showing estimates for the model uncertainty in each slope unit and (*a2*, *b2*, *c2*, *d2*, *e2*, *f2*) success rate curve

To evaluate the influence of land use change on LS zonation, the resulting models were applied using the 1954 land use map. This has been performed computing the percentage of 1954 land use classes for each slope-unit and applying the 2009 model result. Figure 3 shows results of single models prepared using the same grouping variable but changing the land use variables. For each classification model (LDA, QDA, LR), Fig. 3 portrays on the left, the susceptibility zonation prepared using the 2009 land use map and on the right the zonation obtained applying the same model results but considering the 1954 land use map.

The model uncertainty for each slope unit (Fig. 3a1, b1, c1, d1, e1, f1) was computed adopting a “bootstrapping” re-sampling technique (Efron 1979; Davison and Hinkley 2006). For the LDA, QDA, and LR models, 200 model runs were performed, each time varying the selected slope units. Descriptive statistics for the probability (susceptibility) estimates, including the mean (*μ*) and the standard deviation (*σ*), were obtained from the ensembles of the model runs. The relative plots show two standard deviations (2*σ*) of the susceptibility estimates (*y*-axis) against their mean value (*μ*) (*x*-axis) (Rossi et al. 2010). Inspection of the plots reveals interesting similarities and few differences. For all the classification models the measure of variation, 2*σ*, is low for slope units classified as highly susceptible (probability ≥0.80) and as largely stable (probability <0.20), indicating that the models identified consistently these slope units as stable or unstable. The scatter in the model estimate becomes larger for intermediate values of the susceptibility, suggesting not only that the models were incapable of satisfactorily classifying the terrain as stable or unstable for these terrain units, but also that the obtained estimates were highly variable, and hence, less reliable (Guzzetti et al. 2006b). The success rate curves (Fig. 3a2, b2, c2, d2, e2, f2) provide a quantitative indication of the ability of the susceptibility model to match (“fit”) the known distribution of landslides in the study area (Chung and Fabbri 2003). Curves are prepared plotting the total area of known landslides in each susceptibility class with the percentage area of the susceptibility class. The diagrams show the percentage of the study area ranked from most to least susceptible (*x*-axis) subdivided in five classes as the LS maps.

### Exploiting Different Thresholds to Establish Stable/Unstable SU

**Fig. 4:**
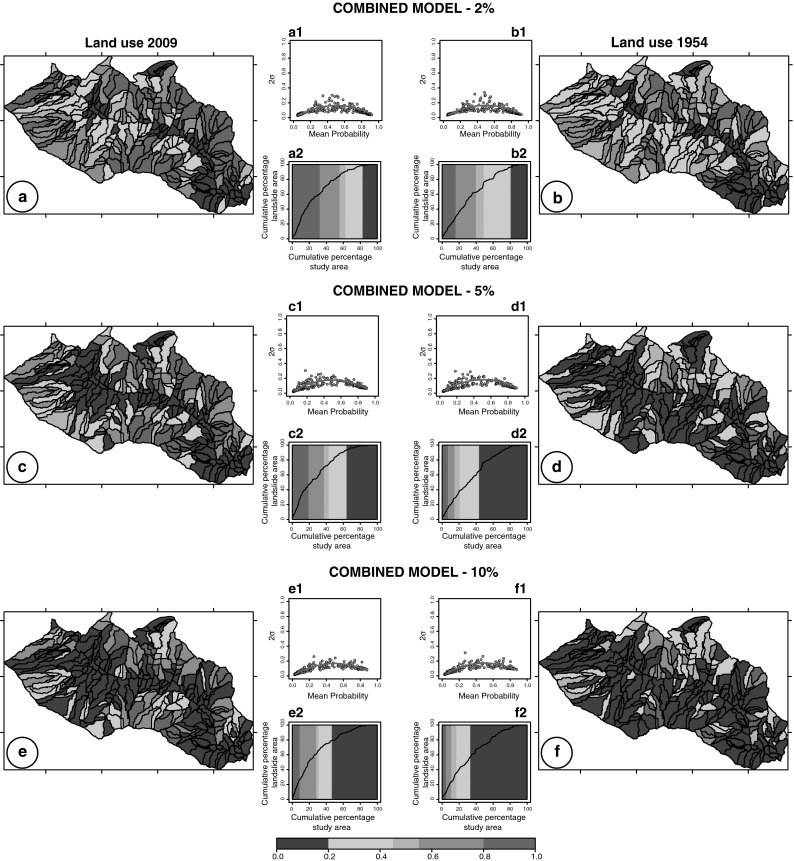
Results of CM landslide susceptibility models prepared using threshold of 2, 5, and 10 % to subdivide the SU considered free of landslides and the SU containing landslides. On the right models prepared using the 2009 land use (**a**, **c**, **e**) and on the left the 1954 land use map (**b**, **d**, **f**) with the predicted LS in five unequally spaced classes; (*a1*, *b1*, *c1*, *d1*, *e1*, *f1*) plot showing estimates for the model uncertainty in each slope unit and (*a2*, *b2*, *c2*, *d2*, *e2*, *f2*) success rate curve

To test the dependence of the model results on the threshold used to establish if a slope unit contained or was free of landslides, we computed the same set of classification models (LDA, QDA, LR, CM), using different empirical thresholds. In particular, we evaluated models using additional thresholds of 5 and 10 % to subdivide the SU considered free of landslides and the SU containing landslides. The upper panel of Fig. 4 shows the susceptibility zonation obtained from the “combined model” (CM) resulting from the single models shown in Fig. 3, whereas the other two panels are the zonation from the CM models, prepared using different thresholds (5 and 10 %). Results of the models obtained using different thresholds are summarized in Table 2 where the output obtained using the 2009 and the 1954 land use maps are listed.

**Table 2:** Results of the CM models (Fig. 4) obtained using different thresholds to subdivide the SU considered free of landslides and containing landslides

	Land use	TP	TN	FP	FN	Total	*A*_ROC_	Sensitivity	Specificity
2 %	2009	36.13	40.34	11.76	11.76	76.47	0.87	0.75	0.77
	1954	31.09	42.86	9.24	16.81	73.95	0.84	0.65	0.82
5 %	2009	26.05	54.20	8.40	11.34	80.25	0.87	0.70	0.87
	1954	13.03	57.98	4.62	24.37	71.01	0.83	0.35	0.93
10 %	2009	18.49	63.87	7.14	10.50	82.36	0.88	0.64	0.90
	1954	9.66	65.55	5.46	19.33	75.21	0.81	0.33	0.92

*TP* true positive, *TN* true negative, *FP* false positive, *FN* false negative

**Fig. 5:**
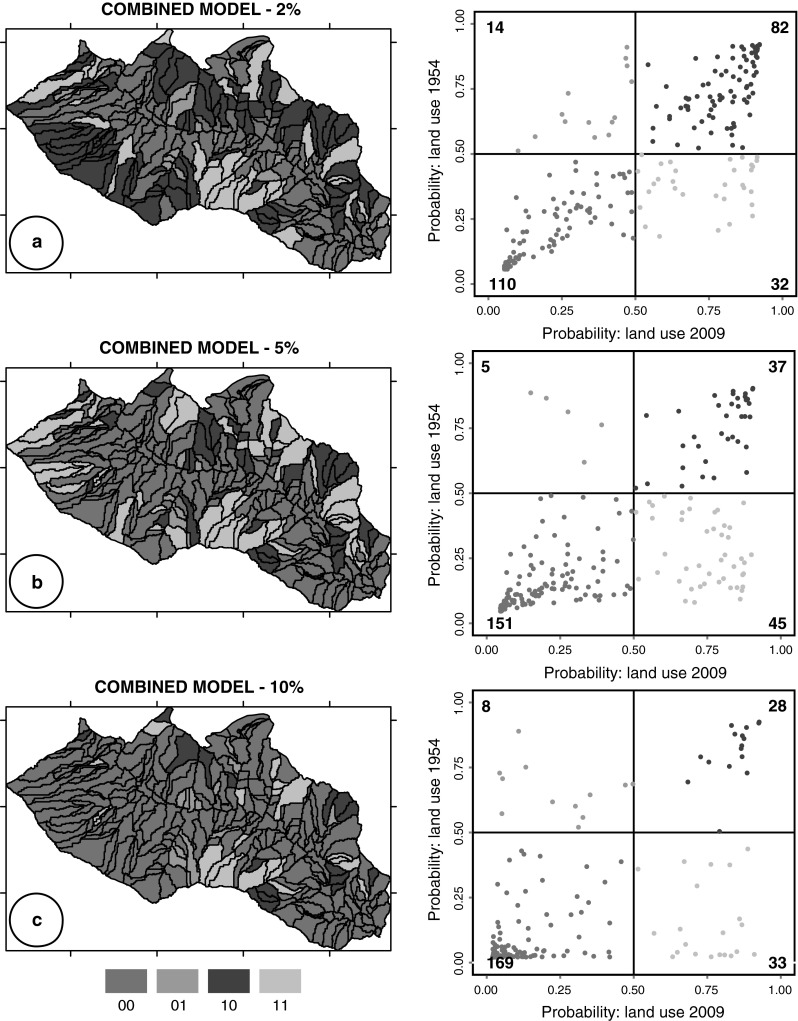
pp-plots and maps show for each slope unit of the CM models (Fig. 4), the difference between the probability values obtained using the 2009 and the 1954 land use maps. In the pp-plots, *dark green* and *dark pink* *points* represent SU classified as stable or unstable in both models whether *light green* and *light pink points* SU with different classification. *Numbers* in the pp-plots show the count of points (Color figure online)

To evaluate the agreement and the difference between the models prepared using the 2009 and the 1954 land use maps, for each threshold (2, 5, and 10 %) we computed a pp-plot (probability–probability plot) comparing for each slope unit the probability value obtained for the CM zonation. Figure 5 illustrates pp-plots and maps showing for each slope unit the difference between the susceptibility models prepared using the 2009 and the 1954 land use maps.

### Land Use Scenarios

To estimate the possible effect of new land use changes on LS, we have designed different scenarios obtained changing the original 2009 land use distribution. Assuming an increase in the forested areas, we have considered three types of changes computed at the slope unit scale resulting in the following scenarios: (i) 75 % decrease in the pasture extent (Scenario 1); (ii) 75 % reduction of both pasture and cultivated areas (Scenario 2); and (iii) 75 % decrease in bare soil where the slope-unit mean angle was greater than 15° associated with 75 % decrease in pasture areas (Scenario 3). A fourth scenario was prepared assuming the effect of a forest fire in the south-west part of the area, where we simulated a reduction of the forested cover and an increase in bare soil (Scenario 4).

**Fig. 6:**
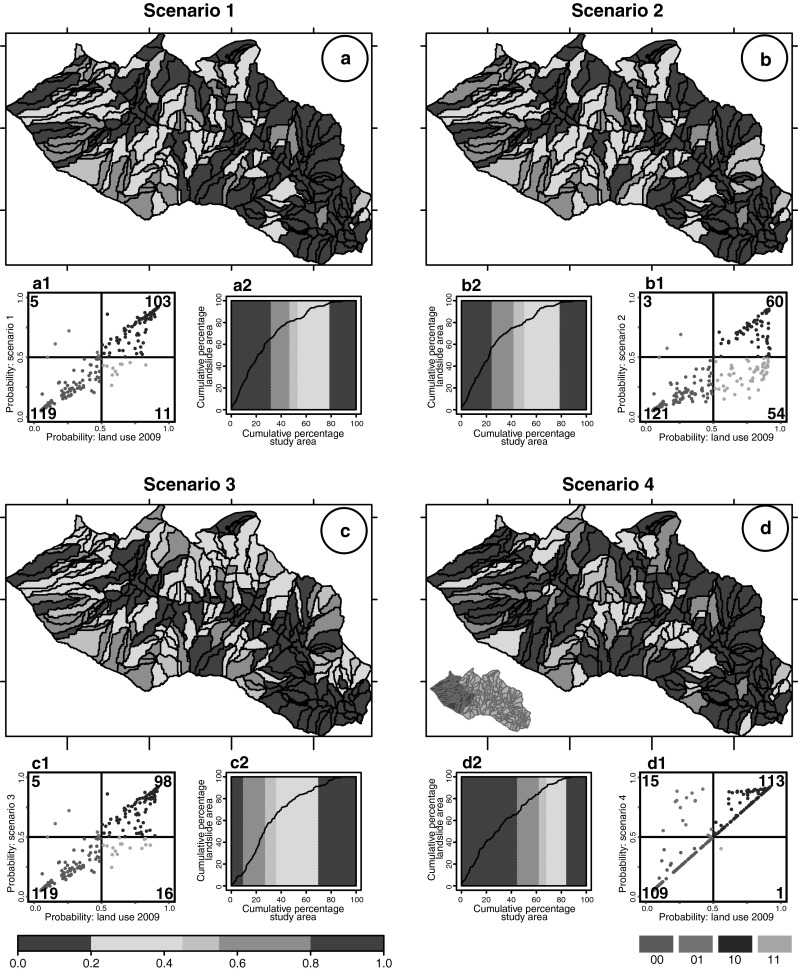
Results of CM landslide susceptibility models prepared using different land use scenario (see text). **a**–**d** Map with the predicted LS in five unequally spaced classes (see legend); (*a1*, *b1*, *c1*, *d1*) pp-plots showing for each slope-unit the difference between the probability value obtained using the 2009 and the new land use scenario (see caption of Fig. 5); (*a2*, *b2*, *c2*, *d2*) success rate curve

For each scenario, Fig. 6 shows: (i) the CM LS zonation, (ii) a pp-plot where the susceptibility calculated for each scenario is compared with the result of the susceptibility model prepared with the 2009 land use, and (iii) a success rate curve measuring the fitting performance of the LS model.

## Discussion

In this paper, we focus on the influence of land use change on LS zonation, exploiting different modeling strategies, analyzing the models’ performance and quantifying the difference between the outputs. We have concentrated our attention on the evaluation of the impact of land use change in a period of about 60 years on landslide spatial occurrence for a small study area located in the Briga catchment. Changes in land use distribution were evaluated comparing maps prepared for two different years. The comparison between the land use maps (Fig. 2) reveals that in more than 50 years the area was influenced by an increase in bare soils to the detriment of forested areas. In the study area, the forest decreased more than 15 %, whereas bare areas, cultivated zones, and urban areas increased about 9, 3, and 3 %, respectively (Table 1). Figure 2 allows visualizing the expansion of urban areas related to the development of new residential zones and the extension of the road network. To verify the effect of land use change, different scenarios were constructed, assuming in three simulations a general increase in forested areas and in a fourth scenario a strong reduction of the forested cover localized in a portion of the basin, considering the ground effect of a fire event.

To understand and quantify the influence of land use change on the susceptibility zonation we performed the following tests: (1) we prepared single models with different classification techniques (LDA, QDA, and LR) (Fig. 3) and we exploited their results, adopting a regression schema in a combined model (CM); (2) we computed single and combined models (LDA, QDA, LR, and CM), using different empirical thresholds to establish if a slope unit contained or was free of landslides (Figs. 4 and 5); and (3) we computed single and combined models simulating different land use scenarios (Fig. 6).

In the first test, to understand the effect of land use change on the susceptibility zonation, we prepared the models using as explanatory variables five land use classes, the presence/absence of pre-event landslides and six morphometric variables obtained from the 5-m DEM and from the slope map (range, mean, standard deviation computed for each slope unit). Figure 3 allows the visual comparison of the susceptibility estimates obtained by the three single models adopting a 2 % threshold to distinguish stable and unstable SU. LDA and LR provided very similar predictions covering the entire range of susceptibility (probability) value whereas the QDA resulted in a higher number of SU classified as unstable. Inspection of the plots showing measures of the model uncertainty (2*σ*) versus the mean probability (*μ*), for each slope unit, reveals that LDA and LR models are characterized by a smaller variability than the QDA model. This confirms, as already argued by Rossi et al. (2010), that the QDA zonation is affected by the largest uncertainty for the SU classified between 0.2 and 0.8, compared to the LDA and LR zonation. However, on average the QDA model uncertainty is lower because the number of SU classified in the two extreme susceptibility classes is higher (Fig. 3e, e2). Zonation maps obtained with the same models (LDA, QDA, and LR) but using the 1954 land use map show similar results with significant reduction in the number of unstable SU. Success rate curves reveal a decrease in the model fitting performance when using the 1954 land use map, due to a reduction of slope units classified as unstable and an increase in stable terrain. In particular, the expansion of bare soil to the detriment of forested areas in the 56 years from 1954 to 2009, determined a general increase in the susceptibility.

These results confirm that single and combined statistically based models are useful and appropriate tools to prepare, evaluate, and compare LS zonation. LS models prepared adopting statistical approaches are quite common where sufficient thematic and environmental information is available. In the literature, in fact several different examples have been described, most of them evaluating the performance skill but very few assessing models uncertainty and prediction skill (Guzzetti et al. 1999; Wang et al. 2005; Kanungo et al. 2009; Pardeshi et al. 2013).

In a second test, we have analyzed the same models (LDA, QDA, LR, and CM), using different empirical thresholds to subdivide the SU considered free of landslides and containing landslides. Figure 4 shows the susceptibility to landslide estimates obtained by the combined models (CMs) adopting a threshold of 2, 5, and 10 %. The threshold increase changes the ratio between stable/unstable SU in favor of stable SU. Moreover, the comparison between the models obtained with the 2009 and the 1954 land use maps indicates that adopting different thresholds the decrease in the number of SU classified as unstable is confirmed (Table 2). In particular, comparing the results obtained for the 2009 versus the 1954 soil maps, a decrease in TP and FP and an increase in TN and FN can be observed. In addition, the overall percentage of mapping units correctly classified by the susceptibility models and the *A*_ROC_ values decrease (area under receiver operating characteristic: a quantitative measure of the model performance. Fawcett 2006; Mason and Graham 2002). This decrease can be explained by a poorer performance of the models when using the 1954 land use map, due to low values of landslide probability occurrence (stable LS classes) in SU affected by slope failures triggered by the 2009 event.

The difference between the zonation obtained with the 1954 and the 2009 land use maps is shown for each slope-unit using pp-plot (probability–probability plot) in Fig. 5. The pp-plot allows us to determine how closely two data sets (in this case the two models) agree. If the two probabilities are similar, the points should form an approximate straight line (i.e., they should be aligned along the bisector of the plot starting from the plot origin), whereas the deviations from this line indicate difference between the models. In our study area, the agreement/disagreement provides indications of the difference between models due to the influence of land use change on LS zonation. In case of agreement between the two models, the land use contribution/control is negligible and the land use change does not affect the zonation. In the graphs, the points below the line aligned along the bisector of the plot, represent SU classified by the 1954 land use model with lower susceptibility values. In particular, the light pink points represent SU classified as unstable when using the 2009 land use map and stable using the 1954 map. The number of the light pink points changes adopting the different thresholds: when using the 2 % threshold, 32 SU (13.4 %) are classified as stable, when using the 5 % threshold, 45 SU (18.9 %) and when using the 10 % threshold, 33 SU (13.8 %). These figures indicate that higher extent of the forested coverage in the 1954 land use map reduces the instability of the terrain. Inspection of Table 3 illustrates the comparison between the CM models prepared using the 2 % threshold, and reveals that the number of unstable SU (probability value greater than 0.55), decrease from 45.4 to 37.8 % when computing the LS zonation with the 1954 land use, while stable SU (probability value <0.45) increase from 47.1 to 55.5 %. The difference is more evident when considering the areal extension of the SU in each susceptibility class: in fact the unstable area decreases from 50.7 to 33.9 % and the stable area increases from 39.0 to 52.2 %.

**Table 3:** Percentage of slope-units in the susceptibility classes computed for the different susceptibility zonation

	<0.20 (%)	0.20–0.45 (%)	0.45–0.55 (%)	0.55–0.80 (%)	>0.80 (%)
Land use 2009	28.2 (15.3)	18.9 (23.7)	7.6 (10.2)	18.9 (24.5)	26.5 (26.19)
Land use 1954	29.0 (16.5)	26.5 (35.65)	6.7 (13.8)	23.1 (18.8)	14.7 (15.1)
Scenario 1	31.1 (21.4)	21.4 (25.8)	6.3 (6.2)	15.5 (14.7)	25.5 (31.8)
Scenario 2	31.1 (21.4)	22.7 (28.5)	8.0 (8.4)	16.8 (17.4)	21.4 (24.3)
Scenario 3	39.5 (30.4)	30.7 (34.0)	7.1 (8.5)	17.6 (17.5)	5.0 (9.6)

The first digit is the percentage of number of SU, the second the percentage of the area (maps and success rate graphs shown in Figs. 4, 6). In the table, we consider only the model results obtained with the 2 % threshold

Although we have considered a small test area, we think our findings are significant and encouraging, because not only they confirm but also quantify the positive effect of forested cover on slope stability. These results can be used to evaluate the consequences of land use change on landslide vulnerability and risk. For environmental planning, regional and municipality authorities should consider the important role of vegetation on slope stability, regulating the clear-cutting of trees, avoiding widespread deforestation, and implementing slope inspection and maintenance at local and basin scale. Woody vegetation, particularly trees, in fact can stabilize hill slopes modifying the soil moisture regime through evapotranspiration processes and providing root cohesion to the soil mantle (Sidle and Ochiai 2006; Ghestem et al. 2011).

In the third test, to evaluate the effect of different land use distributions, we have prepared susceptibility zonation using four different land use scenarios. The maps in Fig. 6 confirm how land use changes affect significantly the number and the areal extent of unstable/stable slope. Moreover, inspection of Table 3 reveals that the increase in stable SU is highly dependent on the land use distribution. The number of SU with a probability value greater than 0.55, is reduced from 45.4 to 22.6 % while stable SU (probability value <0.45) increase from 47.1 to 70.2 %. SU classified as uncertain remain stable. Inspection of Table 3 reveals a clear diminution of the probability values for the first three scenarios with the more optimistic situation related to an enlargement of forest and a reduction of bare soil (Scenario 3). For each scenario pp-plots in Fig. 6 show with the pink points the number of unstable SU turned to stable and with light green points the stable SU turned to unstable. This result confirms the general trend described above.

When assuming a detrimental environmental situation, as a forest fire (Scenario 4), the model output reveals an increase in the number unstable SU and their areal extent. The pp-plot shows a linear distribution of the SU not affected by the change (points along a straight line of the graph) and a set of point located above the line showing an increase in the probability of the SU classified as unstable. Other studies have investigated the effect of forest fire on slope stability from different points of view and using other techniques. Cannon and Gartner (2005) have described for example the physical processes by which post-wild-fire slope failures initiate in different settings whereas deWolfe et al. (2008) have evaluated the effectiveness of erosion control methods at reducing sediment movement in drainage basins burned by wildfire.

## Conclusions

This work proposes to evaluate and quantify the effect of land use change over a period of almost 60 years on the LS zonation in a small study area located at the outlet of the Briga catchment (Messina, Italy). In landslide research literature, it is recognized that forested areas favor terrain stability, but the problem has often been analyzed exploiting physically based models or focusing only on the erosion phenomena. In this paper, we have prepared and described different statistical models to investigate the influence of land use change. The models show an overall variation in the susceptibility zonation: in particular there is a decrease in unstable SU when we consider the 1954 land use distribution that can be justified by the minor extent of bare soils with respect to the forested areas. Other land use change scenarios have been investigated, considering an increase in forested areas to confirm the strong relationship between forest cover and slope stability.

To limit the uncertainty of the analysis, we have prepared susceptibility models, without the use of geologic and other thematic environmental data with the purpose of emphasizing and quantifying the effect of land use on slope stability. Moreover, we have used only DEM-derived variables and land use maps because we think that elevation information is often available and land use maps can be obtained quite easily exploiting aerial photos and/or satellite images. Although this approach is general and does not consider detail and very local land use and/or vegetation information, it proves effective and useful in the evaluation of the effect of land use modification on slope stability. For this reason, we believe that a similar approach can be applied to investigate the impact of land use distribution over larger areas or an entire basin characterized by a similar setting.

This result can be used to evaluate the consequences of land use change on landslide vulnerability and risk. The proposed approach carried out at slope scale combined with local and detailed analysis, could be effective to evaluate the potential effects of different soil cover types on land use planning and slope instability management. In particular, local settings, plant species, and their characteristics should be considered in detail since they play a major role in the soil reinforcement and slope stability (Ghestem et al. 2014).

## References

[CR1] Ardizzone F, Basile G, Cardinali M, Casagli N, Del Conte S, Del Ventisette C, Fiorucci F, Garfagnoli F, Gigli G, Guzzetti F, Iovine G, Mondini AC, Moretti S, Panebianco M, Raspini F, Reichenbach P, Rossi M, Tanteri L, Terranova O (2012). Landslide inventory map for the Briga and the Giampilieri catchments, NE Sicily, Italy. J Maps.

[CR2] Brabb EE (1984) Innovative approaches to landslide hazard mapping. In: Proceedings 4th international symposium on landslides, Toronto, vol 1, pp 307–324

[CR3] Brown CE (1998). Applied multiple statistics in geohydrology and related sciences.

[CR4] Bruschi VM, Bonachea J, Remondo J, Gómez-Arozamena J, Rivas V, Barbieri M, Capocchi S, Soldati M, Cendrero A (2013). Land management versus natural factors in land instability: some examples in northern Spain. Environ Manag.

[CR6] Cannon SH, Gartner JE (2005) Wildfire-related debris flow from a hazards perspective. In: Jakob M, Hungr O (eds.), Debris-flow Hazards and Related Phenomena. Springer Praxis Books, 363-385. doi 10.1007/3-540-27129-5_15

[CR7] Carbone S, Messina A, Lentini F, Barbano MS, Grasso D, Di Stefano A et al (2008) Note Illustrative della Carta Geologica d’Italia alla Scala 1:50.000. Foglio 601 Messina-Reggio di Calabria. Servizio Geologico d’Italia, APAT-Regione Siciliana, vol 1, pp 1–179. SELCA, Firenze (in Italian)

[CR8] Carrara A, Cardinali M, Detti R, Guzzetti F, Pasqui V, Reichenbach P (1991). GIS Techniques and statistical models in evaluating landslide hazard. Earth Surf Process Landform.

[CR9] Carrara A, Cardinali M, Guzzetti F, Reichenbach P, Carrara A, Guzzetti F (1995). GIS technology in mapping landslide hazard. Geographical information systems in assessing natural hazards.

[CR10] Carrara A, Guzzetti F, Cardinali M, Reichenbach P (1999). Use of GIS technology in the prediction and monitoring of landslide hazard. Nat Hazards.

[CR11] Chung C-JF, Fabbri AG (2003). Validation of spatial prediction models for landslide hazard mapping. Nat Hazards.

[CR12] Clemen RT (1989). Combining forecasts: a review and annotated bibliography. Int J Forecast.

[CR13] Cox DR (1958). The regression analysis of binary sequences. J R Stat Soc B (Methodol).

[CR14] Cruden DM, Varnes DJ (1996) Landslide types and processes. In: Turner AK, Schuster RL (eds) Landslides, investigation and mitigation. Transportation Research Board Special Report 247. Transportation Research Board, Washington, DC, pp 36–75

[CR15] Davison AC, Hinkley D (2006) Bootstrap methods and their applications, 8th edn. Cambridge Series in Statistical and Probabilistic Mathematics. Cambridge University Press, Cambridge. ISBN-13: 9780521574716

[CR16] deWolfe VG, Santi PM, Ey J, Gartner JE (2008). Effective debris flow mitigation at Lemon Dam, LaPlata County, Colorado. Geomorphology.

[CR17] Efron B (1979). Bootstrap methods: another look at the jackknife. Ann Stat.

[CR18] Fawcett T (2006). An introduction to ROC analysis. Pattern Recognit Lett.

[CR19] Fisher RA (1936). The use of multiple measurements in taxonomic problems. Ann Eugen.

[CR20] Ghestem M, Sidle RC, Stokes A (2011). The influence of plant root systems on subsurface flow: implications for slope stability. Bioscience.

[CR21] Ghestem M, Veylon G, Bernard A, Vanel Q, Stokes A (2014). Influence of plant root system morphology and architectural traits on soil shear resistance. Plant Soil.

[CR22] Glade T (2003). Landslide occurrence as a response to land use change: a review of evidence from New Zealand. Catena.

[CR23] Goswami R, Mitchell NC, Brocklehurst SH (2011). Distribution and causes of landslides in the eastern Peloritani of NE Sicily and western Aspromonte of SW Calabria, Italy. Geomorphology.

[CR24] Greenway DR, Anderson MG, Richards KS (1987). Vegetation and slope stability. Slope stability.

[CR25] Guzzetti F, Carrara A, Cardinali M, Reichenbach P (1999). Landslide hazard evaluation: a review of current techniques and their application in a multi–scale study, Central Italy. Geomorphology.

[CR26] Guzzetti F, Reichenbach P, Cardinali M, Galli M, Ardizzone F (2005). Probabilistic landslide hazard assessment at the basin scale. Geomorphology.

[CR27] Guzzetti F, Galli M, Reichenbach P, Ardizzone F, Cardinali M (2006). Landslide hazard assessment in the Collazzone area, Umbria, central Italy. Nat Hazards Earth Syst Sci.

[CR28] Guzzetti F, Reichenbach P, Ardizzone F, Cardinali M, Galli M (2006). Estimating the quality of landslide susceptibility models. Geomorphology.

[CR29] Kanungo DP, Arora MK, Sarkar S, Gupta RP (2009). Landslide susceptibility zonation (LSZ) mapping—A Review. J South Asia Disaster Stud.

[CR50] Karsli F, Atasoy M, Yalcin A, Reis S, Demir O, Gokceoglu C (2009) Effects of land-use changes on landslides in a landslide-prone area (Ardesen, Rize, NE Turkey). Environ Monit Assess 156:241–255. doi:10.1007/s10661-008-0481-5 10.1007/s10661-008-0481-5 18780152

[CR30] Mao Z, Yang M, Bourrier F, Thierry Fourcaud T (2014). Evaluation of root reinforcement models using numerical modelling approaches. Plant Soil.

[CR31] Marchesini I, Alvioli M, Rossi M, Santangelo M, Cardinali M, Reichenbach P, Ardizzone F, Fiorucci F, Balducci V, Mondini AC, Guzzetti F (2012) WPS tools to support geological and geomorphological mapping. In: OGRS 2012—Open Source Geospatial Research & Education Symposium—October 24–26 2012, Yverdon-les-Bains, Switzerland. http://2012.ogrs-community.org/2012_posters/WPS%20tools%20to%20support%20geological%20and%20geomorphological%20mapping.pdf. Accessed 20 Aug 2014

[CR32] Mason SJ, Graham NE (2002). Areas beneath the relative operating characteristics (ROC) and relative operating levels (ROL) curves: statistical significance and interpretation. Q J R Meteorol Soc.

[CR33] Maugeri M, Motta E, Iai S (2011). Effects of heavy rainfalls on slope behavior: the October 1, 2009 Disaster of Messina (Italy). Geotechnics and earthquake geotechnics towards global sustainability, geotechnical, geological and earthquake engineering.

[CR34] Meusburger K, Alewell C (2008). Impacts of anthropogenic and environmental factors on the occurrence of shallow landslides in an alpine catchment (Urseren Valley, Switzerland). Nat Hazards Earth Syst Sci.

[CR35] Michie D, Spiegelhalter DJ, Taylor CC (eds) (1994) Machine learning, neural and statistical classification. http://www.amsta.leeds.ac.uk/~charles/statlog/. Accessed 5 Aug 2014

[CR36] Mondini AC, Guzzetti F, Reichenbach P, Rossi M, Cardinali M, Ardizzone F (2011). Semi-automatic recognition and mapping of rainfall induced shallow landslides using satellite optical images. Remote Sens Environ.

[CR37] Pardeshi SD, Autade SE, Pardeshi SS (2013). Landslide hazard assessment: recent trends and techniques. SpringerPlus.

[CR53] Richards JA, Jia X (1999) Remote sensing digital image analysis. Springer, Berlin

[CR38] Rocchini D, Metx M, Frigeri A, Delucchi L, Marcantonio M, Neteler M (2012). Robust rectification of aerial photographs in an open source environmental. Comput Geosci.

[CR39] Rossi M, Guzzetti F, Reichenbach P, Mondini AC, Peruccacci S (2010). Optimal landslide susceptibility zonation based on multiple forecasts. Geomorphology.

[CR40] Sidle RC, Ochiai H (2006) Landslides: processes, prediction, and land use. American Geophysical Union, Water Resources Monograph 18, Washington, DC

[CR41] Stokes A, Norris JE, van Beek LPH, Bogaard T, Cammeraat E, Mickovski SB, Jennern A, Di Iorio A, Fourcaud T, Stokes A, Mickovski SB, Cammeraat E, van Beek R, Nicoll BC, Achim A, Norris JE (2008). How vegetation reinforces soil on slopes. Slope stability and erosion control: ecotechnological solutions.

[CR42] Van Beek LPH, Van Asch TWJ (2004). Regional assessment of the effects of land-use change on landslide hazard by means of physically based modelling. Nat Hazards.

[CR43] Van Den Eeckhaut M, Poesen J, Van Gils M, Van Rompaey A, Vandekerckhove L (2009) How do humans interact with their environment in residential areas prone to landsliding? A case-study from the Flemish Ardennes. In: Proceedings of the international conference on “landslide processes: from geomorphologic mapping to dynamic modelling,” Strasbourg, France, 6–7 February 2009, pp 19–24

[CR44] Vanacker V, Vanderschaeghe M, Govers G, Willems E, Poesen J, Deckers J, De Bievre B (2003). Linking hydrological, infinite slope stability and land-use change models through GIS for assessing the impact of deforestation on slope stability in high Andean watersheds. Geomorphology.

[CR45] Venables WN, Ripley BD (2002) Modern applied statistics with S, 4th edn. Springer, Berlin. ISBN 0-387-95457-0

[CR46] Wang H, Liu G, Xu W, Wang G (2005). GIS-based landslide hazard assessment: an overview. Prog Phys Geogr.

[CR47] Yi J, Bin Y, Jingtao C, Jianwu H, Gaoliao J (2010) Analysis of landslides susceptibility to different land use patterns in Enshi. In: Proceedings of the 2nd conference on environmental science and information application technology, 17–18 July 2010, Wuhan, China

